# Is the standard dose of amoxicillin-clavulanic acid sufficient?

**DOI:** 10.1186/2050-6511-15-38

**Published:** 2014-07-21

**Authors:** Michiel Haeseker, Thomas Havenith, Leo Stolk, Cees Neef, Cathrien Bruggeman, Annelies Verbon

**Affiliations:** 1 Department of Medical Microbiology, Maastricht University Medical Centre, Maastricht, the Netherlands; 2 Department of Clinical Pharmacy, Maastricht University Medical Centre, Maastricht, the Netherlands; 3 Care and Public Health Research Institute (CAPHRI), Maastricht, the Netherlands; 4 Department of Internal Medicine, Erasmus Medical Centre, Rotterdam, the Netherlands; 5 Present address: Maastricht University Medical Centre, P. Debyelaan 25, PO Box 58006202 AZ Maastricht, the Netherlands

**Keywords:** Amoxicillin, Clavulanic acid, Pharmacokinetics, Age

## Abstract

**Background:**

The pharmacodynamic (PD) efficacy target of amoxicillin is 40% time above the minimal inhibition concentration (40%T > MIC). Recent studies of other antibiotics have shown that PD-efficacy targets are not always reached. The aim of this study was to evaluate the percentage of hospitalised patients, using amoxicillin/clavulanic acid intravenously (iv), that reach the pharmacodynamic efficacy target 40%T > MIC. Additionally, the association of demographic anthropomorphic and clinical parameters with the pharmacokinetics and pharmacodynamics of amoxicillin were determined.

**Methods:**

In serum of 57 hospitalised patients amoxicillin concentrations were measured using high performance liquid chromatography. Patients were older than 18 years and most patients had an abdominal infection. The standard amoxicillin/clavulanic acid dose was 4 times a day 1000/200 mg iv. Pharmacokinetic parameters were calculated with maximum *a posteriori* Bayesian estimation (MW\Pharm 3.60). A one-compartment open model was used. Individual dosing simulations were performed with MW\Pharm.

**Results:**

In our study population, the mean (±SD) age was 67 (±16) years and the mean clearance corrected for bodyweight was 0.17 (±0.07) L/h/kg. Only, 65% of the patients reached the proposed amoxicillin 40%T > MIC with amoxicillin/clavulanic acid for bacterial MICs of 8 mg/L. A computer simulated increase of the standard dose to 6 times daily, increased this percentage to 95%. In this small study group 40%T > MIC was not associated with clinical or microbiological cure.

**Conclusion:**

A substantial proportion of the hospitalised patients did not reach the 40%T > MIC with the standard dose amoxicillin/clavulanic acid for a bacterial MIC of 8 mg/L. Therefore, we suggest increasing the standard dose of amoxicillin/clavulanic acid to 6 times a day in patients with severe Enterobacteriaceae infections.

**Trial registration:**

Trial registration number: NTR1725 16^th^ march 2009.

## Background

In vitro and animal studies have shown that β-lactam antibiotics for Gram-positive bacteria and Gram-negative bacteria are effective when the percentage time above the minimal inhibition concentration (%T > MIC) of the unbound serum concentration is more than 35-40%. Maximal effects are reached with %T > MIC above 60-70% [1-3]. Only sparse pharmacokinetic/pharmacodynamic (PK/PD) data are estimated in human clinical studies. Pharmacokinetic analysis of amoxicillin/clavulanic acid has mostly been done in healthy individuals [4,5]. In human clinical studies amoxicillin/clavulanic acid has been found to cure *Streptococcus pneumoniae* and *Haemophilus influenza* infections clinically and microbiologically when %T > MIC was ≥40% [1]. To our knowledge, there are no human pharmacodynamic efficacy studies for Enterobacteriaceae.

Amoxicillin/clavulanic acid is a commonly used broad spectrum antibiotic. Clavulanic acid extends the spectrum of amoxicillin to β-lactamase producing strains, such as *E. coli*, *K. pneumoniae*, *H. influenzae* and *S. aureus*. Clavulanic acid has very little intrinsic antibacterial effect. Clavulanic acid irreversibly inhibits β-lactamase, protecting amoxicillin [6]. Therefore, amoxicillin is measured for determining the PD-efficacy target. Susceptibility patterns of Enterobacteriaceae have changed over time, and recent studies have shown that efficacy targets of other antibiotics, such as ciprofloxacin [7,8], ceftazidim [9] and gentamicin [10] are not reached in a significant percentage of patients. In the EUCAST rationale document the target attainment rates for different doses of amoxicillin for different MICs based on Monte Carlo simulations are reported [11]. Amoxicillin blood levels have not been measured in these simulations. The aim of this study was to measure the amoxicillin serum concentrations in hospitalised patients using amoxicillin/clavulanic acid intravenously (iv) and to determine if the efficacy target of 40%T > MIC was reached. Additionally, we have investigated the association of demographic anthropomorphic and clinical parameters with pharmacokinetics and pharmacodynamics of amoxicillin.

## Methods

### Study design

Patients above 18 years of age treated with amoxicillin/clavulanic acid iv and hospitalised at the Maastricht University Medical Centre (MUMC), a 715 bed university hospital, were included from January 2010 until October 2010. Amoxicillin blood levels were measured in residual samples drawn for routine assays. Patients with at least two blood samples available were included and blood samples taken during or within 0.5 hours after infusion were excluded. Amoxicillin/clavulanic acid (Sandoz, Holzkirchen, Germany) was started at the discretion of the attending physician, either empirically or as therapy for bacteria susceptible to amoxicillin/clavulanic acid. The standard dose of amoxicillin/clavulanic acid was 1000/200 mg 4 times a day and 1000/200 mg 2 times a day was prescribed when the creatinine clearance (CLcr) was 10–50 ml/min. Amoxicillin/clavulanic acid 1000/200 mg/100 mL infusion was infused in 30 minutes. Demographic and clinical data, such as age, gender, weight, temperature, co-medication, length of hospital stay, time of administration of amoxicillin/clavulanic acid and laboratory parameters, such as, serum creatinine, C-reactive protein (CRP) were retrieved from the electronic patient file (iSoft, the Netherlands). Clinical outcome was defined by CRP normalisation, number of admission days, and discharge from the hospital, time to defeveresence and microbiological cure. CLcr was calculated with the Cockcroft-Gault formula using the lean body mass.

This study was registered at the Dutch Trial Register (NTR 1725) [12] and was approved by the Medical Ethical Committee of the Maastricht University Medical Centre (MEC 08-4-063). All data in this study were analysed anonymously and amoxicillin blood levels were measured in residual samples drawn for routine assays. Therefore, no consent was required from the patient. This is in agreement with the Medical Research Involving Human Subjects Act, the code for proper use of human tissue as formulated by the Dutch Federation of Medical Scientific Societies and the policy of the Medical Ethics Committee of the Maastricht University Medical Centre.

### HPLC analysis

A simple, fast and specific method for measuring amoxicillin serum levels has been validated for linearity, precision, accuracy and stability, following the guidelines for industry bioanalytical method validation recommended by the Food and Drug Administration (FDA) [13]. In short, serum samples were precipitated with perchloric acid 3%. A reverse phase high pressure liquid chromatography (RP-HPLC) method was used. The calibration range was 10 to 200 mg/L. Six quality controls (8, 20, 40, 60, 80 and 160 mg/L) were tested. The intra- and inter-assay variability was within 7.5%. The relationship between plasma concentration and sd was fitted with a polynomial of second order [14]. With this polynomial the lower limit of quantification with a precision of 20% has been calculated and was 0.8 mg/L [15]. All our measured concentrations were above 0.8 mg/L.

### Pharmacokinetic analysis

Pharmacokinetic parameters of amoxicillin in individual patients were calculated with maximum *a posteriori* (MAP) Bayesian estimation program (computer program MW/Pharm 3.60, Mediware, the Netherlands). A one-compartment open pharmacokinetic model was used. With MAP Bayesian estimation [16,17] all patient characteristics and measured amoxicillin concentrations are fitted on an existing population model [4,5]. With two concentrations per patient individual pharmacokinetic parameters can be adequately calculated with MAP Bayesian estimation [17]. With these individual fitted pharmacokinetic parameters, dosing simulations were made to adjust the dose individually; this MAP Bayesian estimation is standard procedure in laboratories which provide therapeutic drug monitoring service. A population kinetic model from our population was not made. The free fraction of amoxicillin was fixed at 80%. The %T > MIC was determined with the formula of Turnidge; %T > MIC = ln(Dose/VdxMIC) × T½/ln(2) × 100/dosing interval [18]. Individual dosing simulations were performed with MW-Pharm (3 times 1000–2000 mg, 4 times 2000 mg, 5 times 1000–2000 mg, 6 times 1000–2000 mg).

### Microbiological analysis

Identification of the causative bacterium (ID) and antibiotic susceptibility testing (AST) were performed with the Becton Dickinson Phoenix™ Automated Microbiology System (Franklin Lakes, New Jersey, USA) using the ID/AST Combo panels UNMIC/ID53 and NMIC/ID75 for Gram negative bacteria and the AST panel PMIC-58 for Gram positive bacteria. In urine cultures the lowest MIC detectable was 0.5 mg/L by the UNMIC/ID53 panel (standard clinical care). MIC values were not determined by the Becton Dickinson Phoenix™ for Streptococcal spp. and Anaerobes. Susceptibility testing for Streptococcal spp. and Anaerobes was done with the disc diffusion method.

### Statistical analysis

Metric variables were tested for normality of distribution by the Shapiro-Wilk test and presented as mean (±SD). If not, median and ranges were also given. Categorical variables are presented as frequencies and percentages. Univariate analysis on the amoxicillin clearance corrected for bodyweight (CLam/W) with categorical variables was either done by Student *t*-test or by one-way ANOVA. Data analysis was done with SPSS-pc version 16.0. A *P*-value of <0.05 is considered to be statistically significant.

## Results

### Study group

A total of 57 patients with a mean (±SD) of 3 (±0.9) blood samples (median: 2, range 2–5 blood samples) were included. The mean age was 67 (±16) years (median: 69, range 23–93 years) and 70% were male (Table 1). About half of the patients had an abdominal infection, 18% a wound infection and 10% a pneumonia (Table 1).

**Table 1:** Characteristics of 57 hospitalised patients

	**Mean (±SD)**	**Median (range)**
Age in years	67 (±16)	69 (23–93)
Weight in kg	78 (±20)	75 (43–153)
Number of blood levels	3 (±0.9)	2 (2–5)
Amoxicillin iv days	13 (±9)	10 (3–57)
Admission days	27 (±34)	19 (4–188)
Gender	Number (percentage)	
• Male	40 (70%)	
• Female	17 (30%)	
Infection	Number (percentage)	
• Abdominal infection	28 (49%)	
• Wound infection	10 (17.5%)	
• Pneumonia	6 (11%)	
• Urinary tract infection	3 (5%)	
• Other	10 (17.5%)	
Amoxicillin combination	Number (percentage)	
• Monotherapy	44 (78%)	
• Ciprofloxacin	4 (7%)	
• Erythromycin	3 (5%)	
• Gentamicin	3 (5%)	
• Other^a^	3 (5%)	
Co-medication	Number (percentage)	
• None	16 (28%)	
• Cardiovascular	27 (47%)	
• Diabetic mellitus	15 (26%)	
• Immunosuppressive	3 (5%)	
• Other^b^	8 (14%)	

^a^Other: rifampicin, cefuroxim and metronidazol.

^b^Other: haloperidol, levothyroxine, painkillers, anticoagulant.

### Pharmacokinetic and pharmacodynamic analysis

The mean CLam/W was 0.17 (±0.07) L/h/kg (median: 0.16, range: 0.05-0.37 L/h/kg) and the mean volume of distribution corrected for bodyweight (V/W) was 0.31 (±0.07) L/kg (median: 0.30, range: 0.19-0.50 L/kg). The mean volume of distribution (V) was 24 (±5.6) L (median: 24, range: 14–36 L) and the mean elimination half-life (t½) was 1.5 (±0.6) h (median: 1.3, range: 0.6-3.29 h) which is in the same range as described in the EUCAST rationale document [11]. The mean serum creatinine was 90 (±36) μmol/L (median: 90, range: 49–210 μmol/L). Fifty-five (96%) patients received the standard dose of amoxicillin/clavulanic acid 4 times a day 1000/200 mg iv, 1 patient 3 times 1000/200 mg iv and 1 patient 2 times a day 1000/200 mg iv, both because of renal insufficiency. Patients above 70 years had lower CLam/W (*P* = 0.02), Table 2. A significant correlation was found between CLam/W and age (*P* < 0.001).

**Table 2:** **Mean (±SD) CLam/W**^**a**^**, CLcr**^**b**^**, V/W**^**c **^**and T½**^**d **^**for amoxicillin in patients using amoxicillin/clavulanic acid broken down per age group**

**Age group in years**	**N**	**CLam/W in L/h/kg**	**CLcr in mL/min**	**V/W in L/kg**	**T½ in h**
<70	31	0.19 (±0.08)	82 (±25)	0.30 (±0.06)	1.26 (±0.44)
>70	26	0.14 (±0.06)	55 (±19)	0.32 (±0.08)	1.83 (±0.71)
P-value		0.02	<0.01	0.82	<0.01

^a^CLam/W: amoxicillin clearance corrected for bodyweight.

^b^CLcr: creatinine clearance.

^c^V/W: volume of distribution corrected for bodyweight.

^d^T½: amoxicillin half life.

**Figure 1:**
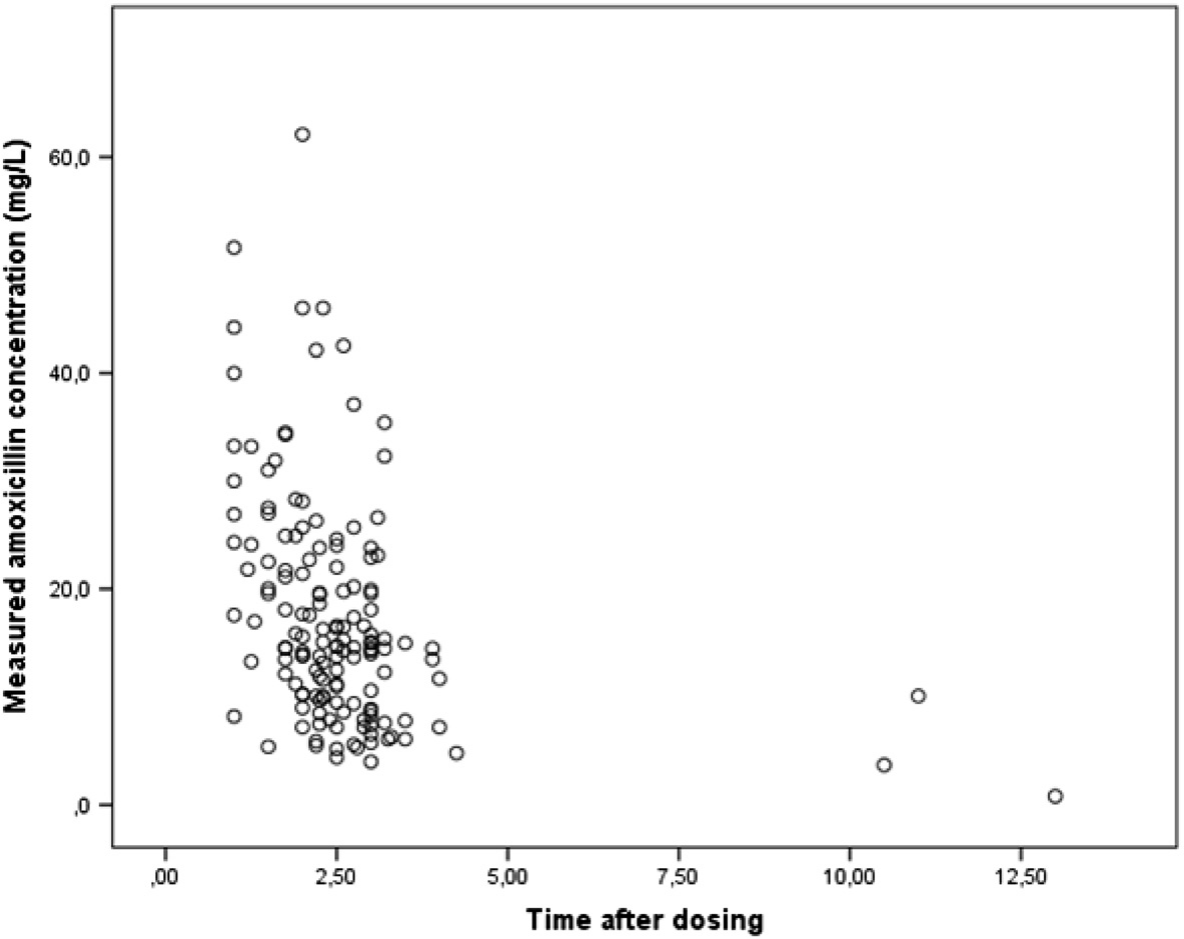
The measured concentrations of amoxicillin plotted against the time after amoxicillin administration.

**Figure 2:**
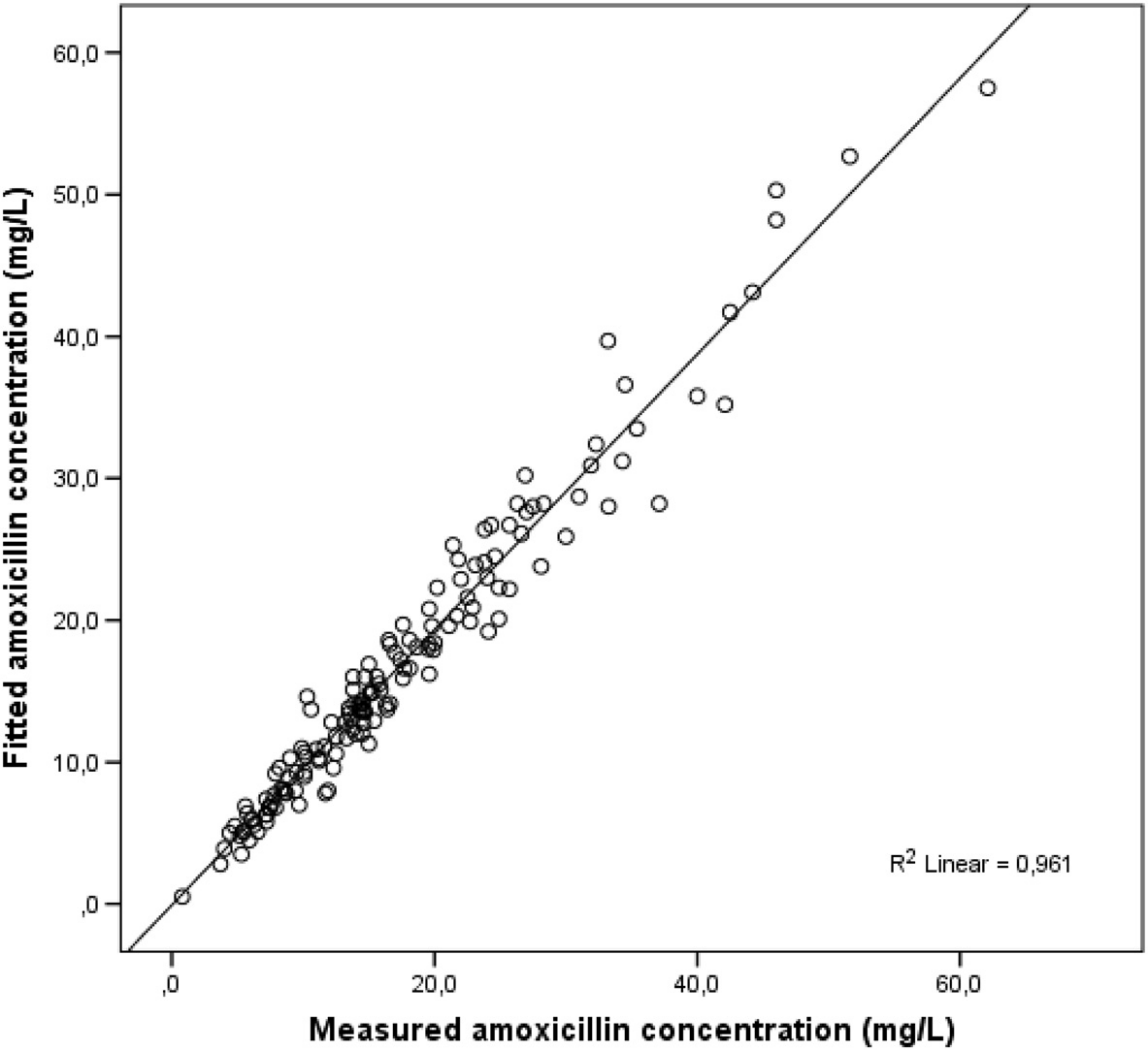
Correlation between amoxicillin actual measured concentration and estimated with maximum a posteriori Bayesian fitting (MW/Pharm 3.60, Mediware, the Netherlands).

**Figure 3:**
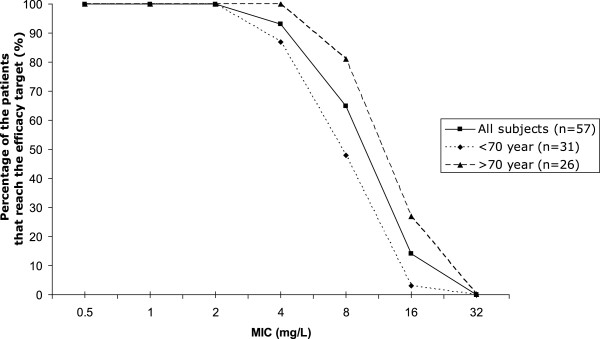
The percentage of patients that reach the 40%T > MIC for different age categories at different MICs.

The measured concentrations of amoxicillin were plotted against the sampling times in Figure 1. There was a good linear correlation (R^2^ = 0.96) between the amoxicillin actual measured concentration and estimated concentration with MAP Bayesian fitting (MW/Pharm 3.60, Mediware, the Netherlands), Figure 2. The amoxicillin efficacy target (40%T > MIC) was reached in 100% of patients with a bacterial MIC ≤ 2 mg/L of Gram negative bacteria, in 93% of patients with a MIC = 4 mg/L and in 65% of patients with a MIC = 8 mg/L (Figure 3). When divided in age categories, arbitrarily set at 70 years; all patients older than 70 years reached the 40%T > MIC with a MIC of 4 mg/L and 87% of the patients younger than 70 years. For a MIC of 8 mg/L, the 40%T > MIC was reached in 81% of the patients older than 70 years and in 52% of the patients younger than 70 years (Figure 3).

### Analysis of influence of co-variates on pharmacokinetic and pharmacodynamic parameters

To determine which co-variates have an effect on the pharmacokinetic parameters of amoxicillin, a univariate analysis was done using a predetermined set of predictors (Table 3). In the univariate analysis CLam/W was related to age, creatinine, CLcr, V/W and 40%T > MIC. Linear correlations were found between CLam/W, creatinine and age (R^2^ 0.327), Vd (R^2^ 0.370) and age (R^2^ 0.227). In the univariate analysis age was not correlated to creatinine (R 0.10, *P* = 0.467) and no linear correlation (R^2^ 0.01) was found.

**Table 3:** Univariate Pearson correlation coefficients between amoxicillin/clavulanic acid, CLam/W and predictors used in this study

	**Univariate CLam/W**	
	**R**	***P*-value**
Creatinine	-0.584	<0.001
Age	-0.476	<0.001
Gender	-0.034	0.812
V/W	-0.608	<0.001
40%T > MIC	-0.424	0.025

CLam/W, amoxicillin/clavulanic acid clearance corrected for bodyweight.

V/W, amoxicillin/clavulanic acid volume of distribution corrected for bodyweight.

### Dosing simulations

**Figure 4:**
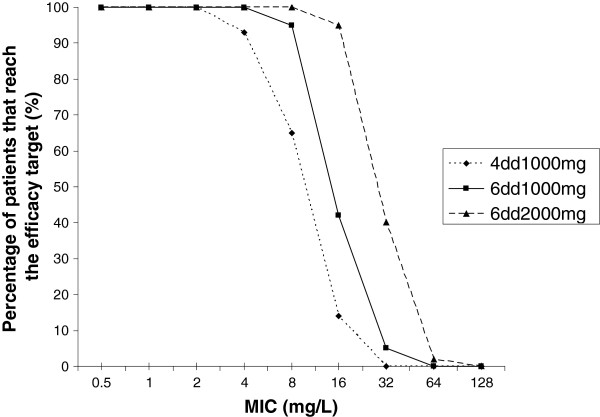
Calculated percentage of patients with 40%T > MIC at different MICs for increasing amoxicillin dosages.

To determine whether increasing the dose of amoxicillin/clavulanic acid would lead to sufficiently high %T > MIC, the %T > MIC was calculated for all patients with increasing doses with the Turnidge formula [18]. When increasing the dosage frequency from 4 times to 6 times a day all patients with bacterial MIC ≤ 4 reach the efficacy target and 95% (54/57) of the patients with bacterial MIC ≤ 8 (Figure 4).

### Microbiological analysis

Sixteen out of twenty one abdominal fluid cultures became positive, 8/10 wound cultures, 7/16 blood cultures, 4/5 urine cultures and 1/2 sputum cultures became positive. Of three patients no cultures were taken. In total thirty-six of the 57 patients (63%) had a positive culture. Of the positive cultures, 30 (83%) were Enterobacteriaceae; *E. coli* (n = 21), *Klebsiella* spp. (n = 6), *Enterobacter* spp. (n = 2) and *Proteus* spp. (n = 1). In three patients two bacteria were isolated. Of the Gram positive cultures, 6 were *Enterococcus* spp, 3 *Staphylococcus aureus* and 1 coagulase negative staphylococcus*.* Forty MIC values were available in 36 patients. Of the isolated Enterobactericeae 17/30 (57%) of had a MIC ≤ 4 mg/L, 4/30 (13%) had a MIC = 8 mg/L and 9/30 (30%) had a MIC ≥ 16 mg/L. Of the isolated Gram positive bacteria 8/10 (80%) of had a MIC ≤ 1 mg/L and 2/10 (20%) had a MIC ≥ 4 mg/L. Clinical cure was reached in 46 patients, 8 patients switched antibiotic therapy and in 3 patients cure was not reached (of which two died). No significant associations were found between 40%T > MIC and defeverescence, CRP decrease or increase, admission days and clinical outcome (data not shown).

## Discussion

In this study, we demonstrate that the efficacy target of 40%T > MIC for amoxicillin/clavulanic acid was reached in 93% of the patients tested when the MIC was 4 mg/L and only in 65% of the patients tested when the MIC was 8 mg/L. In the EUCAST and CLSI criteria Enterobacteriaceae are considered to be susceptible for amoxicillin/clavulanic acid with bacterial MIC ≤ 8 mg/L [11]. High bacterial MICs for amoxicillin/clavulanic acid are an increasing problem in the Netherlands and in Europe [19,20]. To prevent treatment failure for individual patients and to prevent development of antibiotic resistance on population level, increasing the standard dose of amoxicillin/clavulanic acid seems warranted. Dosing simulation showed that increasing the standard dose of amoxicillin/clavulanic acid to 6 times a day 1000/200 mg increased the number of patients reaching 40%T > MIC to 100% for bacterial MIC ≤ 4 and to 95% with bacterial MIC ≤ 8. Continuous iv dosing is an alternative for frequent dosing of time dependent β-lactam antibiotics. Unfortunately, amoxicillin is not very suitable for continuous iv dosing, because of the instability of amoxicillin at room temperature. Therefore, we suggest increasing the dose of amoxicillin/clavulanic acid to 6 times a day in patients with severe Enterobacteriaceae sepsis or intra-abdominal infection.

In general amoxicillin/clavulanic acid is well tolerated. The most frequent adverse drug events are diarrhoea, nausea and vomiting. However, amoxicillin/clavulanic acid is also associated with liver injury, which is estimated to occur from 1 to 1.7 per 10.000 users [21,22]. Clavulanic acid seems to be responsible for the adverse drug reaction, since amoxicillin alone is rarely associated with liver injury and causes less gastrointestinal problems than the combination preparation [23-25]. In vitro pharmacodynamic studies demonstrate that low dose of clavulanic acid suffice and the β-lactamase inhibition of clavulanic acid lasts for 8–12 hours [26,27]. Therefore, increasing the standard dose of amoxicillin/clavulanic acid of 4 times a day 1000/200 mg iv with amoxicillin twice daily 1000 mg iv may be a safe and effective alternative.

CLam/W is correlated with CLcr and the amoxicillin dose is adjusted with to the CLcr. However, other covariates also influence the CLam/W. CLam/W was significantly correlated with age. However, age and creatinine were not correlated to each other, meaning that elderly patients can have both a normal creatinine and a decreased CLam/W. Therefore, the correlation of age with CLam/W seems independent of the creatinine. Furthermore, the 4 patients that did not reach the efficacy target with bacterial MIC = 4 were all young patients with excellent clearance. Our measured attainment results are lower than those calculated attainment results in the EUCAST rationale document, in which Monte Carlo simulations were used to calculate the target attainment rates (40%T > MIC) of different dosing regimens (from 500 mg 3 times a day to 2 g 4 times a day) for different bacterial MICs (0.5-32 mg/L) [11]. The target attainment in the EUCAST rationale document for the standard dose (1000/200 mg 4 times a day) is 100% at bacterial MIC ≤ 4 and 75% with bacterial MIC ≤ 8 mg/L [11]. In our real life blood level determination study, these percentages were 93% and 65%, respectively. This difference may be explained by the larger interindividual variability of our population and in particular by a group of younger patients with normal renal clearance (CLcr > 60 mL/min). Remarkably, in the EUCAST rationale document, the interindividual variation is extremely small; t½ is 1.1 (±0.1) h, versus t½ is 1.5 (±0.6) h in our study. The higher t½ in our study may be due to the high mean age of our population.

No significant associations have been found between the target 40%T > MIC and clinical outcome. As expected, our study population was too small and too heterogeneous. A large number of patients are needed to draw conclusions for this endpoint. Moreover, in an in vitro study, ceftazidim has been shown to be maximally effective when 40%T > MIC was reached for concentrations four times the MIC or higher [28]. In our study, the target of 40%T > 4 × MIC was only reached in 100% for low MICs (≤1 mg/L), but never for bacterial MICs of 4 and 8 mg/L. Our study was not designed to isolate a large number of bacterial MICs and therefore only a limited number of clinical bacterial MICs were available in our study. Taken together a large clinical PK/PD study of amoxicillin/clavulanic acid is needed with microbiological and clinical cure endpoints to establish the association between clinical endpoints and the efficacy target 40%T > MIC.

## Conclusions

The current standard dose of amoxicillin/clavulanic acid 4 times a day 1000/200 mg iv is too low to reach the 40%T > MIC for bacterial MIC of 8 mg/L in a high percentage of patients. To prevent treatment failure for individual patients and to prevent development of antibiotic resistance on population level, we suggest increasing the standard dose of amoxicillin/clavulanic acid to 6 times a day in patients with an Enterobacteriaceae sepsis or intra-abdominal infection.

## Abbreviations

AST: Antibiotic susceptibility testing; CLam/W: Amoxicillin clearance corrected for bodyweight; CLcr: Creatinine clearance; CRP: C-reactive protein; ID: Identification of the causative bacterium; iv: Intravenous; MAP: Maximum *a posteriori*; MIC: Minimal inhibition concentration; MUMC: Maastricht University Medical Centre; PK: Pharmacokinetic; RP-HPLC: Reverse phase high pressure liquid chromatography; SD: Standard deviation; T: Time; V/W: Volume of distribution corrected for bodyweight.

## Competing interests

The authors declare that they have no competing interests.

## Authors’ contributions

MH, TH and LS carried out the data analysis. CB, LS, CN an AV participated in design of the study. MH, TH, LS, CN, CB and AV drafted the manuscript. All authors have read and approved the final version manuscript.

## Pre-publication history

The pre-publication history for this paper can be accessed here:

http://www.biomedcentral.com/2050-6511/15/38/prepub
